# The Therapeutic Potential of Umbilical Cord Mesenchymal Stem Cells in Mice Premature Ovarian Failure

**DOI:** 10.1155/2013/690491

**Published:** 2013-08-13

**Authors:** Shufang Wang, Ling Yu, Min Sun, Sha Mu, Changyong Wang, Deqing Wang, Yuanqing Yao

**Affiliations:** ^1^Blood Transfusion Department, the General Hospital of the People's Liberation Army, Beijing 100853, China; ^2^Department of Obstetrics and Gynecology, First Affiliated Hospital of the General Hospital of the People's Liberation Army, Beijing 100048, China; ^3^Department of Obstetrics and Gynecology, Tangdu Hospital, Fourth Military Medical University, Xi'an 710038, China; ^4^Department of Obstetrics and Gynecology, the General Hospital of the People's Liberation Army, Beijing 100853, China; ^5^Tissue Engineering Research Center of Academy of Military Medical Sciences, Beijing 100850, China

## Abstract

Mesenchymal stem cells, which are poorly immunogenic and have potent immunosuppressive activities, have emerged as promising cellular therapeutics for the treatment of several diseases. Mesenchymal-like cells derived from Wharton's Jelly, called umbilical cord matrix stem cells (UCMSCs), reportedly secrete a variety of cytokines and growth factors, acting as trophic suppliers. Here, we used UCMSCs to treat premature ovarian failure (POF). Ovarian function was evaluated by ovulation and the number of follicles. Apoptosis of the granulosa cells (GC) was analyzed by TUNEL staining. We found that after transplantation of the UCMSCs, apoptosis of cumulus cells in the ovarian damage model was reduced and the function of the ovary had been recovered. The sex hormone level was significantly elevated in mice treated with UCMSCs. The number of follicles in the treated group was higher than in the control group. Our results demonstrate that UCMSCs can effectively restore ovary functionality and reduce apoptosis of granulosa cells. We compared the RNA expression of the UCMSCs treated group with the POF model and wild-type control group and found that the UCMSC group is most similar to the wild-type group. Our experiments provide new information regarding the treatment of ovarian function failure.

## 1. Introduction

Premature ovarian failure (POF), also referred to as hypergonadotropic hypogonadism, is classically defined as 4–6 months of amenorrhea in women under the age of 40 and is associated with menopausal levels of serum gonadotropins (FSH > 40 IU/L) and hypoestrogenism. POF prevents women from being able to conceive and may also be associated with other medical problems, such as blood clots, osteoporosis, and heart disease [1]. 

The cause of POF is complex. Some patients have abnormal ovary development because of genetic disorders, such as fragile X, Turner syndrome, and some autosomal genes mutation [2]. Some cases of POF are attributed to autoimmune disorders [3]. Also chemotherapy and radiation treatments for cancer can sometimes cause ovarian failure [4]. There are two basic categories of premature ovarian failure: one in which there are few to no remaining follicles, and in the second, there are an abundant number of follicles. In the first situation, the causes include genetic disorders [5], chemotherapy, radiation to the pelvic region, surgery, and so on; however, in most cases, the cause is unknown. In the second case, a frequent cause is autoimmune ovarian disease, which damages maturing follicles but leaves the primordial follicles intact. Currently, there are no proven treatments that restore normal functionality to a woman's ovaries [6]. One of the most common treatments for women with premature ovarian failure is hormone replacement therapy (HRT), but HRT has been shown to increase the risk of blood clots in the veins, ovarian cancer, and breast cancer [7, 8]. 

Mesenchymal stem cells (MSCs) have been shown to differentiate into various cell types both *in vivo* and *in vitro*. They are found in embryonic and extraembryonic tissues, as well as in adult organs [9]. Several reports indicate that cells from Wharton's Jelly (WJ), the main component of the umbilical cord extracellular matrix, are multipotent stem cells, expressing markers of bone marrow mesenchymal stem cells (BM-MSCs). The human umbilical cord was found to be a new source of stem cells and can be used for cell therapy [10]. UCMSCs express the MSC markers CD29, CD44, CD73, CD90, and CD105 and do not express CD31, CD45, and HLA-DR [11]. UCMSCs can differentiate into several mesodermal cell types. Some groups have started using stem cells to restore the functionality of failed neuron in animals [12, 13]. Additionally, UCMSCs result in almost no immune response [14] and can be used to treat graft versus host disease (GVHD) [15]. The low expression of human leukocyte antigen (HLA) major histocompatibility complex (MHC) Class I and the absence of MHC Class II molecules in UCB-derived MSCs indicate the presence of immune evasion, even in allogeneic transplantations [16–18]. There is considerable optimism that immune suppression and the anti-inflammatory properties of MSCs will be useful in many conditions, such as graft versus host disease, solid organ transplants, and pulmonary fibrosis [19–21]. 

Researchers have used mesenchymal stem cells to repair damaged ovaries in rats [22, 23]. This is further supported by clinical reports linking stem cell transplantations to the spontaneous and as-yet unexplained recovery of ovarian function and natural fertility in some women rendered prematurely menopausal by high-dose cyclophosphamide (CTX). However, whether human UCMSCs can help mice damaged ovaries recover and the optimal dose at which they should be administered have not yet been determined. 

## 2. Methods

### 2.1. Animals

Specific pathogen-free (SPF-) grade CD1 (ICR) mice were purchased from Vital River. The mice were housed in the animal facility at the Academy of Military Medical Sciences and studied at 8–10 weeks of age. All studies followed procedures consistent with the Academy of Military Medical Sciences' guide for the care and use of laboratory animals. 

### 2.2. Cell Isolation and Culture

Full-term umbilical cords (UCs) were received following UC blood removal. We used the mechanical dissociation and explanted culture method. Whole UCs were manually dissected into smaller sections (5-6 grams UC tissue per isolation) and plated in polystyrene tissue culture flasks with L-DMEM supplemented with 10% fetal bovine serum (Gibco, Carlsbad, CA, USA) and 1% penicillin/streptomycin (Gibco, Carlsbad, CA, USA) for 7 days in a 37°C humidified environment with 5% CO_2_ [24]. After around 10 days the first colonies of UCMSCs, the UCMSCs can be seen. At confluence, the cells were harvested with 0.25% trypsin-EDTA.

### 2.3. Phenotype Analysis by Flow Cytometry

The UCMSCs surface marker expression was analyzed at passage six by flow cytometry, using fluorescein isothiocyanate (FITC)-conjugated human monoclonal antibodies against the following: CD105, CD90, CD29, CD44, human leukocyte antigen (HLA)-DR, and Phycoerythrin (PE-) conjugated human antibodies against CD34. For flow cytometry analysis, adherent cells were detached by treatment with 0.25% trypsin-EDTA, neutralized with FBS-containing culture medium, and disaggregated into single cell by pipetting. The cells were incubated with mAbs for 30 min at 4°C, washed twice with PBS, resuspended in 0.5 mL PBS, and immediately analyzed using an FACS Calibur flow cytometer (Becton Dickinson). At least 2 × 10^5^ cells were used for each sample, and Cell Quest software was used for data analysis. 

### 2.4. POF Model

The mice were randomly divided into three groups of 15 mice each, namely, the WT group, the POF group, and the UCMSCs group. In the latter two groups, the mice received a daily dose of intraperitoneal CTX injection (50 mg/kg) for 15 consecutive days to establish POF models of chemotherapy-induced ovarian damage. 

### 2.5. Cell Transplantation

Mice in the UCMSC group were injected intravenously with 1 × 10^6^ hUCMSCs in 100 *μ*L PBS according to previous study [25–27], and those in the WT and POF group were injected with 100 *μ*L PBS alone. The injections were repeated once on the following day. For cell tracking studies, the cells were washed with PBS, incubated with the fluorescent CM-Dil (Roche) for 5 min at 37°C and 15 min at 4°C, washed twice with PBS, and resuspended in PBS for a final volume of 10^7^ cells/mL. The recovery rate of this procedure was 80–90% of the viable cells.

### 2.6. Mice Superovulation

Female ICR mice were superovulated by an intraperitoneal injection of 5 IU of pregnant mare serum gonadotropin followed, 48 h later, by intraperitoneal injection of 5 IU of human chorionic gonadotropin (hCG). Fully grown GV oocytes were collected from 4- to 8-week-old ICR female mice 44–48 h after equine chorionic gonadotropin injection. The MII oocytes were collected from the oviducts of female mice, administered with equine chorionic gonadotropin and human chorionic gonadotropin. Parthenogenetic activation of MII oocytes was performed by culturing the oocytes in calcium-free CZB medium supplemented with 10 mM strontium and 1 mg/mL cytochalasin B (CB).

### 2.7. Hematoxylin and Eosin Staining of Ovary Slices

After one-week transplantation, the ovaries of three group mice were collected. The mouse ovaries were fixed in phosphate-buffered saline containing 4% formaldehyde, and routine paraffin-embedded sections were produced. The ovaries of three groups were collected five days after treatment. The 4 *μ*m paraffin-embedded sections were rehydrated using xylene and a graded alcohol series and stained with hematoxylin and eosin. 

### 2.8. Hormone Examination

Before the experiment, 1 mL of blood was collected in the diestrus phase and the serum was restored in a procoagulant tube (BD) for determination of the baseline levels of estradiol (E_2_). The concentrations of estradiol in the ovarian homogenate were determined by chemiluminescence techniques.

### 2.9. Apoptosis Detection

*In Situ* Cell Death Detection Kit, POD (Roche) was used for detection of cell apoptosis in the mouse ovaries. TUNEL (terminal deoxynucleotidyl transferase-mediated dUTP nick end-labeling) is a method of choice for rapid identification and quantification of the apoptotic cell fraction. The *In Situ* Cell Death Detection Kit is designed to detect fragmented DNA histochemically by TUNEL (TdT-mediated dUTP Nick End Labeling). The fluorescein-labeled nucleotides are incorporated *in situ* onto the 3′ ends of DNA fragments, allowing histologic localization and detection of individual apoptotic cells.

Briefly, the tissue sections were washed in xylene and ethanol (absolute, 95%, 90%, 80%, and 70%, diluted in double distilled water), suspended in PBS, and incubated for 15–30 min at 21°C–37°C with proteinase K (10–20 *μ*g/mL in 10 mM Tris/HCL, pH 7.4–8). The slides were rinsed twice in PBS, and the area around the sample was dried. Then, 50 *μ*L of TUNEL reaction mixture was added to the samples, which were incubated for 60 min at 37°C in a humidified atmosphere in the dark. The slides were rinsed 3 times with PBS. We then dyed the cell nucleus with Hoechst 33324 for 15 min and washed 3 times with PBS.

### 2.10. RNA Microarray Analysis

RNA was isolated from mouse ovaries using Trizol (Invitrogen) using standard methods.

Labeling and hybridization were performed at the CapitalBio Company, according to protocols described in the 32 K mouse genome arrays user manual. The data were analyzed using LuxScan 3.0 Image analysis software (CapitalBio company). 

### 2.11. Real-Time Quantitative PCR

Total RNA was extracted from WT, POF, and UCMSC group ovary using Trizol (Life Technologies). Superscript II reverse transcriptase (Life Technologies) was used to generate cDNA using 1 *μ*g of RNA and oligo dT primer, according to the manufacturer's instructions. The primers were designed using Primer Express version 2.0 (Applied Biosystems, Foster City, CA, USA) and are listed in Table 1. The primers used for this study were synthesized by Invitrogen. The qRT-PCR was performed in triplicate and was repeated in at least three separate experiments using the following conditions. Reaction mixtures contained 12.5 *μ*L of SYBR Green I dye master mix (Applied Biosystems), 2 pmoles each of forward and reverse primers, and 5 *μ*L of 100 times diluted cDNA. Thermocycle conditions included initial denaturation at 50°C and 95°C (10 min each), followed by 40 cycles at 95°C (15 s) and 60°C (1 min). Fluorescent data were acquired during each extension phase. After 40 cycles, a melting curve was generated by slowly increasing (0.1°C/s) the temperature from 60°C to 95°C, while the fluorescence was measured. The threshold cycle (CT) was calculated using the Sequence Detector Systems version 1.2.2 (Applied Biosystems) by determining the cycle number at which the change in the fluorescence of the reporter dye (ΔRn) crossed the threshold. To synchronize each experiment, the baseline was set automatically by the software. To rule out DNA contamination in the RNA preparations, the qRT-PCR controls were performed with RNA templates which did not show any amplification.

## 3. Results

### 3.1. Characterizing the UCMSCs

**Figure 1:**
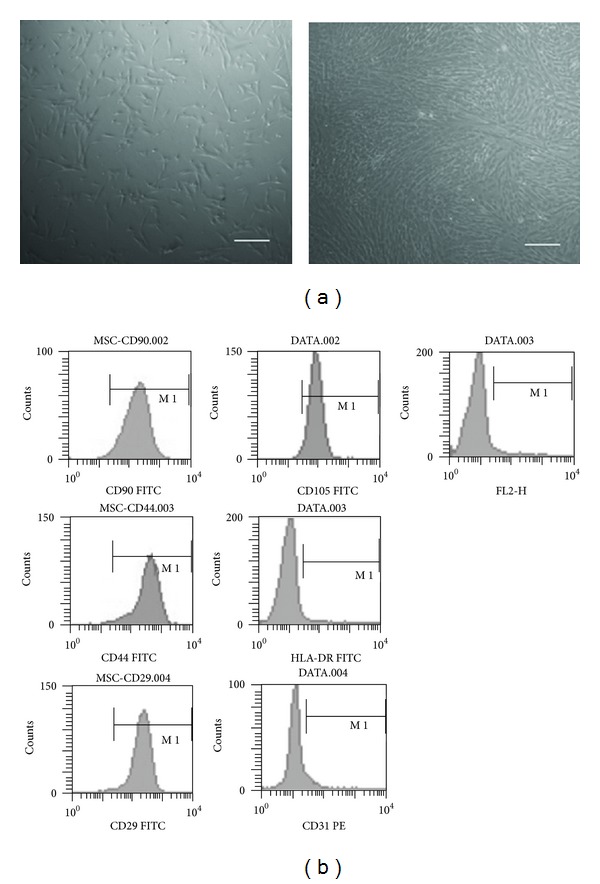
Isolation and identification of UCMSCs. (a) The UCMSCs were isolated from human umbilical cord and showed typical fibroblastic morphology. (b) FCM analysis of UCMSCs. CD90, CD44, CD29, and CD105 were positive and CD31, HLA-DR were negative. Scale bars: 100 *μ*m.

The UCMSCs were harvested and cryopreserved 20–30 days after the initial processing, as time was required for the UCMSCs to migrate from the plated tissue (Figure 1(a)). A minimum of 10 days was required for the first colonies of UCMSCs to appear, and additional 5 days were needed to gain 70–80% confluency, at which point the cells were transferred to 75 cm^2^ flasks. The proliferation rate decreased after 10 passages. 

The hMSCs from different sources displayed a homogenous spindle-shaped population and maintained this morphology during subsequent passages. FACS analysis was employed to identify the surface marker expression at passage 6. The UCMSC culture was shown to be devoid of HLA-DR and CD31. In contrast, a high expression of CD29, CD44, CD90, and CD105 markers were observed (Figure 1(b)). These markers were expressed in over 95% of the population. 

### 3.2. UCMSCs Rescue the POF Ovulation Function

**Figure 2:**
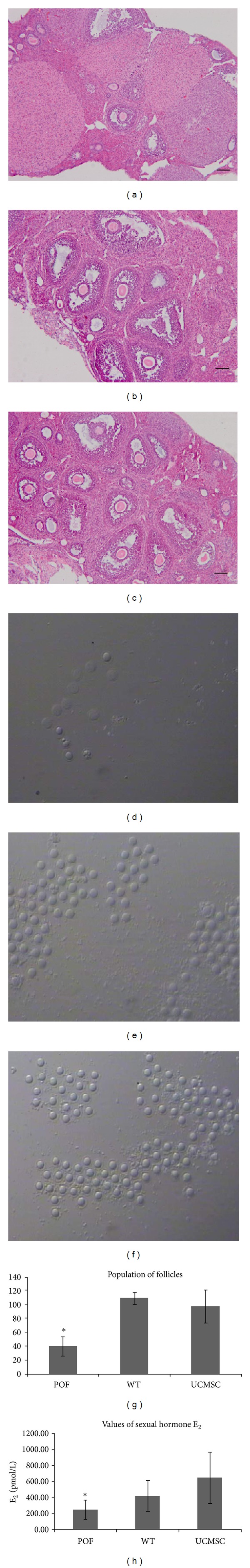
The effect of transplantation. (a, b, c) Hematoxylin eosin staining. (a) Follicles of the POF model. (b) Follicles of the WT group. (c) Follicles of the UCMSCs treated group. (d) Oocytes of the POF model. (e) Oocytes of the WT group. (f) Oocytes of the UCMSCs treated group. (g) Number of follicles of the three groups (POF, WT and UCMSCs treated). Data were means ± SD of numerous experiments. (POF versus WT *P* < 0.01, POF versus UCMSC *P* < 0.01, and UCMSC versus WT *P* > 0.05). (h) The values of sexual hormone E_2_ of the three groups (POF, WT and UCMSCs treated). Data were means ± SD of numerous experiments. (POF versus WT *P* < 0.05, POF versus UCMSC *P* < 0.05, and UCMSC versus WT *P* > 0.05). Scale bars: 100 *μ*m.

Females administered nonlethal doses of CTX (50 mg/kg) for 15 days exhibited premature ovary failure. There were fewer follicles in their ovaries than in the wild-type mice (Figures 2(a) and 2(b)). The wild-type mouse ovary possessed 111 ± 8 follicles, and the POF mouse ovary possessed 39 ± 13 follicles (Figure 2(g)) (POF versus WT *P* < 0.01). The UCMSCs were delivered intravenously. The ovaries were collected one week after treatment, and the presence of normal follicles was determined. There was a slight increase in the weight of the ovaries in mice undergoing cell transplantation. The ovaries from female mice that were injected intravenously through the tail contained the similar number of follicles as the wild-type mice (108 ± 15) (UCMSC versus WT *P* > 0.05) (Figures 2(c) and 2(g)). And ovaries of mice receiving UCMSCs transplantation after chemotherapy possessed more oocyte-containing follicles at various stages of development than POF group (POF versus UCMSC *P* < 0.01) (Figures 2(d), 2(e) and 2(f)).

### 3.3. The UCMSCs Recover Ovary Function in Sex Hormone (E_2_) Levels

Follicular development was assessed in all groups by monitoring E_2_ levels in the serum. One week after MSC transplantation, the serum E_2_ of the therapy group was significantly increased compared with the POF group (*P* < 0.05) without treatment. There was no significant difference between the UCMSC treated group and WT groups (Figure 2(h)).

### 3.4. The UCMSCs Do Not Differentiate into Follicles

The cell-tracking technique may be generally suited to monitoring stem cell homing and engraftment. One week after transplantation, the intravenously injected labeled human UCMSCs could be traced in the ovaries. The labeled stem cells infused into the donor ovaries but did not develop into follicles, including cumulus cells or oocytes (data not show). 

### 3.5. The UCMSCs Can Reduce Apoptosis of the Granulosa Cells

**Figure 3:**
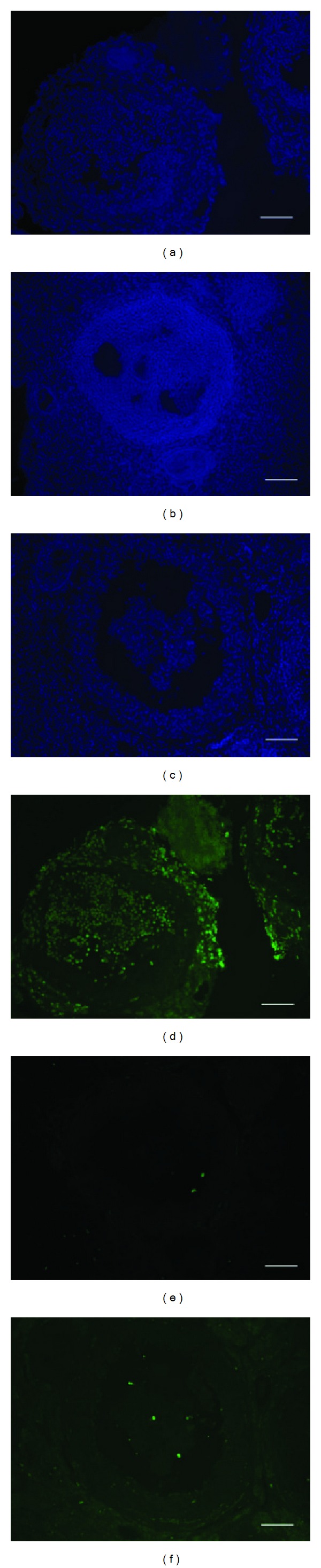
Apoptosis of granulosa cells around follicles. (a, b, c) Green fluorescence indicates the apoptosis of granulosa cells in the three groups (POF, WT, and UCMSCs treated). (d, e, f) Blue fluorescence indicates the nucleus of granulosa cells. Scale bars: 50 *μ*m. The green stained by POD kit. The blue stained by Hoechst.

Following CTX, the granulosa cells and other cells in the ovaries underwent dramatic apoptosis. One week after UCMSCs transplantation, the percentage of TUNEL-positive GCs in the therapy group was significantly lower than that in the POF group (Figure 3). After the UCMSCs therapy, the apoptosis amount is almost the same as the wild-type group. 

### 3.6. The UCMSC Therapy Group RNA Expression Pattern Is Closer to Wild-Type Than to That in the POF Model

**Figure 4:**
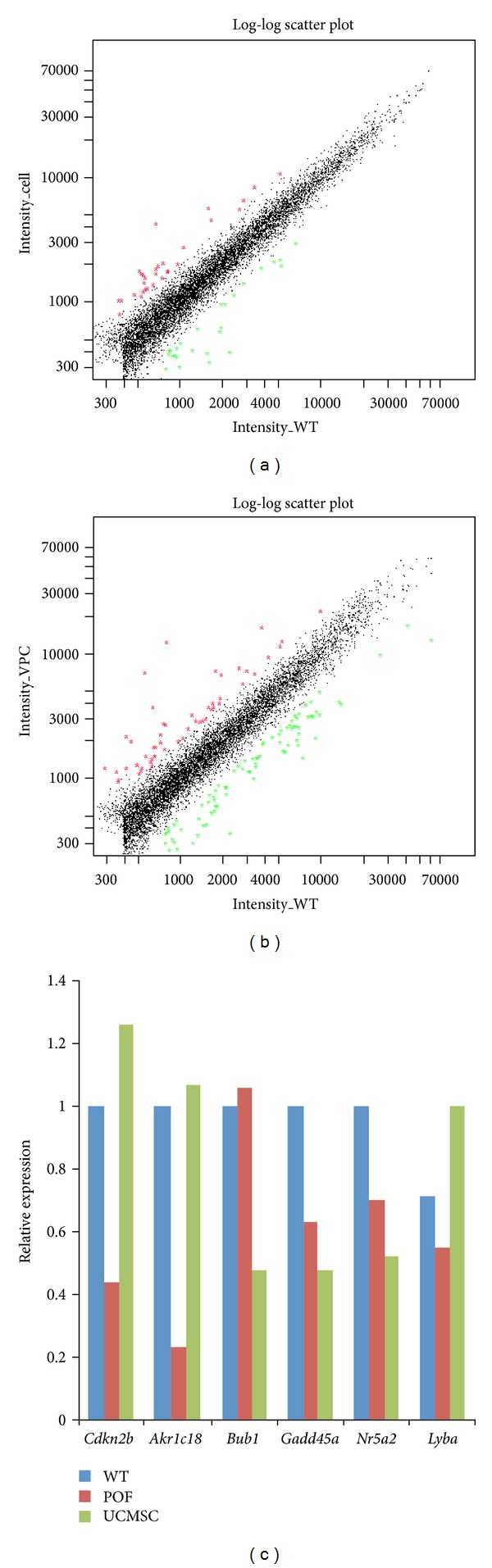
RNA array analyses. (a) UCMSC group has 16 genes higher expression than WT group and 15 genes lower expression than WT group. Red: gene upregulated. Green: gene downregulated. (b) POF group has 33 genes higher expression than WT group and 58 genes lower expression than WT group. Red: gene upregulated. Green: gene down-regulated. (c) Genes relative expression of the three groups.

The RNA expression pattern in the cell therapy and the WT groups was almost the same, only 31 genes different between the UCMSCs transplantation group and the WT group, 16 upregulated and 15 downregulated (Table 2). The POF group had 33 genes more highly expressed than the WT group and 58 genes with lower expression than the WT group (Figure 4 and Table 3). 

**Figure 5:**
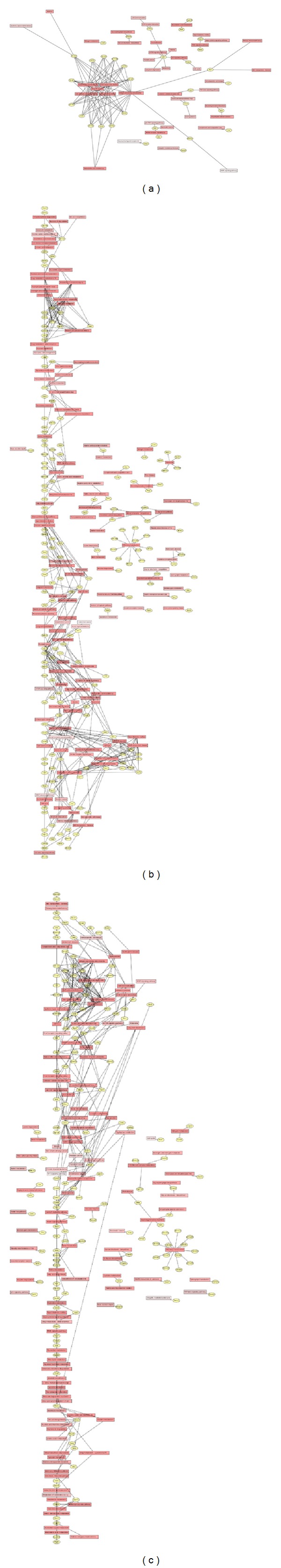
Genes correlation of three pathways. (a) UCMSC-WT gene Pathway Network. (b) POF-UCMSC gene Pathway Network. (c) POF-WT gene Pathway Network.

Comparing the gene expression between the WT and POF groups, we found that a number of pathways were affected, including transcription regulation, the G-protein coupled receptor protein signaling pathway, the MAPK pathway, and the insulin pathway (Figure 5). These pathways are important for follicle and oocyte growth.

In addition to important pathways, there are also some important proteins that can be rescued by UCMSCs. Aldo-keto reductase family 1 members catalyze androgen, estrogen, and prostaglandin metabolism. AKR1C18 expression was very low in the POF model group mice. Existing as a monomer, AKR1C18 catalyzes the NADP+-dependent conversion of progesterone into 20-*α*-dihydroprogesterone, a biologically inactive metabolite [28]. This reaction is thought to be important for luteolysis (the degradation of the corpus luteum), as well as for overall reproductive health and newborn survival in mice. After stem cell therapy, the amount of AKR1C18 increased to normal levels. 

LY6A, also named SCA-1, is a stem cell marker [29]. The mouse ovarian surface epithelium contains a population of LY6A (SCA-1) expressing progenitor cells that are regulated by ovulation-associated factors. The ovarian surface epithelium, a single layer of poorly differentiated epithelial cells, covers the surface of the ovary and is ruptured during ovulation. LY6A, also known as stem cell antigen-1 (SCA-1), has determined that the size of the LY6A-expressing (LY6A+) progenitor cell population is regulated by at least two ovulation-associated factors present in the follicular fluid, and LY6A has identified a population of LY6A+ MOSE progenitor cells on the surface of the ovary that may play a role in ovulatory wound healing. 

The CDKN2B gene encodes a cyclin-dependent kinase inhibitor, also known as p15Ink4b protein, which forms a complex with CDK4 or CDK6 and prevents the activation of the CDK kinases by cyclin D. Thus, the encoded protein functions as a cell growth regulator that inhibits cell cycle G1 progression [30]. The expression of this gene was dramatically induced by TGF beta, which suggested its role in TGF beta-induced growth inhibition. p15INK4B is a potential effector of TGF beta-induced cell cycle arrest. When the ovary is destroyed by CTX, many cells undergo apoptosis. The ovary has to initiate more cell growth to supply follicle development, so the genes involved in cell cycle arrest are downregulated. After cell therapy, the expression of genes involved in cell cycle regulation returned to normal levels.

## 4. Discussion

Stem cells have emerged as a key element of regenerative medicine therapies due to their inherent ability to differentiate into variety of cell phenotypes, thereby providing numerous potential cell therapies to treat degenerative diseases. Although early menopause frequently occurs in female cancer patients after chemotherapy (CTX), bone marrow transplantation (BMT) has been linked to a recovery of ovarian function and fertility in some survivors. Stem cell transplantation has been found to restore the fertilization ability of patients [31], although the mechanism is not known.

UCMSCs have several properties that make them an interesting source of cells for therapeutic use. In our experiments, human UCMSCs could be isolated rapidly in large numbers from 90% of human cords. Thus, UCMSCs cells may be a robust source of MSC-like cells for therapeutic use because they can be frozen/thawed, clonally expanded, engineered to express exogenous proteins, and extensively expanded in culture.

We found that, after transplantation of UCMSCs, the function of the ovary recovered. Previous study has shown that UCMSCs transplanted by intravenous migrate into several organs after hours [32]. And the ovulation of mice needs 4 days, so we chose after one week to measure the UCMSC location and function. Although we found that human UCMSCs successfully recover mouse ovary function, we did not find that the cells differentiate into follicle components. Previous studies have shown that ES-conditioned media can improve oocyte maturation *in vitro* [33]. The UCMSCs are more likely to function through the paracrine pathway. We found that the UCMSC treatment group expressed more proteins involved in the DNA modification epigenetic pathway, the transcription pathway, the protein modification pathway, and cell signaling. UCMSCs quickly repair ovarian damage by granulosa cell growth stimulated by these pathways. The UCMSCs can also increase some ovarian stem cell function proteins, including AKR1C18 and Ly6a (Sca-1). Levels of the cell cycle control protein CDKN2B in the stem cell transplantation group returned to the levels normally observed in the wild-type group. UCMSCs can secrete potent combinations of trophic factors that modulate the molecular composition of the environment to evoke responses from resident cells [34–37].

In addition to accelerating the repair of damaged ovarian cells, UCMSCs dramatically reduce apoptosis of granulosa cells in the developing follicle. Most of the follicles progress to apoptosis after exiting from the arrested stage of the cell cycle. When CTX is administered to the mice, apoptosis increases to an even higher level. After UCMSC treatment, the rate of apoptosis is reduced. Previous studies have shown that stem cell can inhibit apoptosis through secretion of stanniocalcin-1, paracrine factors, improve the oxide response pathway, [38–40]. Therefore, we believe that UCMSCs function primarily by reactivating host oogenesis, which becomes impaired in a reversible manner after CTX (e.g., cell cycle arrest) or by indirect effects of the drug on the microenvironment (niches).

In summary, this study supports the idea that UCMSCs can rescue mouse ovary impairment, without causing immune rejection. In addition to BMT, we showed that hUCMSCs could rescue chemotherapy-induced POF in mice. hUCMSCs possess the immune properties of immune suppression and immune avoidance [41]. Following xenotransplantation of human UCMS cells into immune-competent rats, little to no host immune cell infiltration was observed, suggesting that UCMSCs possess low immunogenicity [42]. We provided evidence demonstrating that, after transplantation of UCMSCs, the functionality of the POF ovary is recovered and improved. 

The potential clinical applications are immediately apparent. Each year millions of women undergo fertility treatments due to age-related loss of oocytes; these treatments offer only modest improvements in fertility. By the middle of the fifth decade, the therapies are largely futile. Furthermore, millions of young women are rendered sterile by chemotherapy-associated oocyte loss. If a viable source of oocyte production remains in these women, there is the potential to restore fertility. The identification of these stem cells gives hope to these women and leads us to ask if fertility restoration is possible. Further understanding and potential manipulation of the adult female germ stem cell niche may provide answers. In addition, at least for high-dose CTX-exposed females who exhibit reduced posttreatment lethality, UCMSCs might also rescue fertility as part of a more global beneficial effect on overall health. 

## Figures and Tables

**Table 1:** The primers were used for the qRT-PCR.

Gene	GB.accession		Primer sequence 5′ → 3′
GAPDH		F	GTTGTCTCCTGCGACTTCA
		R	TGGTCCAGGGTTTCTTACTC
Actin		F	GAGATTACTGCTCTGGCTCCTA
		R	GGACTCATCGTACTCCTGCTTG
Bub1	NM_001005856.1	F	ATGAAGCCACAACGTACCCA
		R	TCCTGCCGTCTACTCCTCTTG
Cdkn2b	NM_007670.4	F	TTTGGGTGGGTGCAGTCAG
		R	TTCCTTGTCGAGCTGGAGGT
Chek1	NM_007691.5	F	TGGATGCGGACAAATCTTACC
		R	ACCAAACCTTCTGGCTGCTC
Gadd45a	NM_007836.1	F	CTCATCCGTGCGTTCTGCT
		R	TCCATTGTGATGAATGTGGGTT
Cdk4	NM_009870.3	F	CAGTCTACATACGCAACACCCG
		R	CAAAGATTTTCCCCAACTGGTC
Cxcr4	NM_009911.3	F	GCTGAAAAGGCAGTCTATGTGG
		R	ACAGGCTATCGGGGTAAAGG
Lhcgr	NM_013582.2	F	ACGCTGACCTACCCTAGCCA
		R	CCCAGCCACTGAGTTCATTCT
Ly6a	NM_010738.2	F	CAATTCTTCCTGGAGCCCTCT
		R	CATGAGCAGCAATCCACAACA
Smad2	NM_010754.5	F	CACCTTCATCACGCCTTGG
		R	TGGGCATCTGCTTTCTTTGA
Nr5a2	NM_030676.3	F	TCAGTTCGATCAGCGGGAGT
		R	TTCACCTGCTCTTGGACACCT
Akr1c18	NM_134066.2	F	CAAGTCCATCGGGGTGTCTAA
		R	GCAGCTTGCTCTGGTTGAGAT
Cyp4f15	NM_134127.1	F	ATGACCCCTTCCGCTTTGA
		R	CGCCACCTTCATCTCGTTC

**Table 2:** The different expression genes between the UCMSCs treated group and the WT group.

Name	Ratio = UCMSCs treated group/wt group	Oligo_id
**Cfd**	**7.5935**	**M300002011**
**Car3**	**6.2399**	**M200000108**
**Adipoq**	**5.0866**	**M200001531**
**Tgtp**	**3.4595**	**M400008751**
**Fbxo39**	**2.7945**	**M300012693**
**Megf10**	**2.7605**	**M400013376**
**Nuf2**	**2.6878**	**M300005239**
**Rmcs2**	**2.6072**	**M200002417**
**Lbp**	**2.5563**	**M400000246**
**Ly6k**	**2.4823**	**M200014514**
**Saa1**	**2.3428**	**M400010894**
**Q4VCP1_MOUSE**	**2.3163**	**M400004214**
**U3**	**2.2201**	**M400015091**
**Chek1**	**2.1561**	**M300008080**
**Tspan11**	**2.1209**	**M200011768**
**D12Ertd647e**	**2.0849**	**M400009945**
*Col6a2 *	*0.4936 *	*M400000370 *
*Lix1 *	*0.4928 *	*M300003775 *
*Sfrs11 *	*0.4562 *	*M400009262 *
*Cyp4f18 *	*0.4557 *	*M400011465 *
*Akap2 *	*0.4443 *	*M400010322 *
*Sectm1a *	*0.438 *	*M200015271 *
*St3gal5 *	*0.4214 *	*M400005399 *
*Gdpd3 *	*0.4141 *	*M200005744 *
*Cyp4f18 *	*0.376 *	*M400000072 *
*Sectm1b *	*0.3312 *	*M200011927 *
*Gpnmb *	*0.2996 *	*M300006890 *
*4930422I07Rik *	*0.298 *	*M300008255 *
*Ptgds *	*0.2978 *	*M200000369 *
*Cxcr4 *	*0.2072 *	*M400014726 *
*Cd7 *	*0.1569 *	*M200001594 *

The different expression genes between the UCMSCs treated group and the WT group. There are 31 genes, 16 genes upregulated and 15 genes downregulated. Bold: upregulated genes. Italic: downregulated genes.

**Table 3:** The different expression genes between the POF group and the WT group.

Name	Ratio = POF group/WTgroup	Oligo_id
**Adipoq**	**9.7237**	**M200001531**
**Actg2**	**9.1892**	**M300000862**
**Fbxo39**	**8.1171**	**M300012693**
**Car3**	**7.243**	**M400010743**
**—**	**6.3766**	**M400011025**
**Fabp4**	**5.857**	**M300005685**
**Car3**	**5.6195**	**M200000108**
**—**	**5.4981**	**M400000320**
**H2-Ea**	**4.2115**	**M300010457**
**—**	**4.0834**	**M400009758**
**Tyrobp**	**3.3287**	**M200014463**
**H2-DMb2**	**2.9302**	**M300010992**
**Glo1**	**2.8122**	**M400009038**
**Tspan11**	**2.6785**	**M200011768**
**—**	**2.6672**	**M400006287**
**Aqp2**	**2.6298**	**M200003832**
**Bcl2a1c**	**2.5787**	**M400004604**
**Cdo1**	**2.5221**	**M200006769**
**Cd74**	**2.4419**	**M400010174**
**H2-T22**	**2.3833**	**M400006288**
**Hbb-b1**	**2.3606**	**M400000660**
**—**	**2.3277**	**M400008839**
**Ier3**	**2.324**	**M200005156**
**—**	**2.3114**	**M400003688**
**2210403B10Rik**	**2.2961**	**M300009216**
**Sars**	**2.237**	**M400011126**
**C3**	**2.224**	**M200003738**
**Drbp1**	**2.1762**	**M400012193**
**Hspb1**	**2.1179**	**M300017926**
**Spcs1**	**2.1146**	**M200006195**
**Folr1**	**2.097**	**M200000812**
**Sfrs5**	**2.0605**	**M400000441**
**Abcb1b**	**2.0557**	**M200016221**
*Slc16a9 *	*0.4928 *	*M200003760 *
*Cp *	*0.4873 *	*M300000454 *
*— *	*0.4805 *	*M400006370 *
*Glipr1l2 *	*0.4764 *	*M200015469 *
*S100a4 *	*0.4727 *	*M400011125 *
*— *	*0.4643 *	*M400009718 *
*— *	*0.462 *	*M400013641 *
*— *	*0.46 *	*M400010667 *
*— *	*0.4533 *	*M300016225 *
*Phf20l1 *	*0.4494 *	*M300010640 *
*Scarb1 *	*0.4413 *	*M300011232 *
*— *	*0.4392 *	*M400001328 *
*— *	*0.4376 *	*M400009125 *
*Tnc *	*0.4353 *	*M400001084 *
*— *	*0.4329 *	*M400013571 *
*— *	*0.4168 *	*M400008229 *
*Atoh1 *	*0.4148 *	*M400010733 *
*— *	*0.4115 *	*M400008154 *
*2610042L04Rik *	*0.4105 *	*M400005704 *
*— *	*0.41 *	*M400008548 *
*Onecut2 *	*0.4098 *	*M300016911 *
*Nr5a2 *	*0.4027 *	*M300005083 *
*— *	*0.4027 *	*M400008507 *
*— *	*0.4004 *	*M300012538 *
*LOC673685 *	*0.3998 *	*M400012723 *
*Slc6a6 *	*0.3969 *	*M300007001 *
*Procr *	*0.3923 *	*M200001248 *
*Ap4b1 *	*0.3854 *	*M200006396 *
*— *	*0.3796 *	*M400010333 *
*4930534B04Rik *	*0.3795 *	*M200007634 *
*Ptgds *	*0.3744 *	*M200000369 *
*— *	*0.3691 *	*M300005163 *
*— *	*0.3686 *	*M400006364 *
*— *	*0.3684 *	*M200002493 *
*— *	*0.3677 *	*M400015154 *
*— *	*0.3655 *	*M400007777 *
*— *	*0.3613 *	*M400005487 *
*— *	*0.3525 *	*M400011042 *
*Decr1 *	*0.3385 *	*M200004880 *
*Ly6a *	*0.3354 *	*M300009411 *
*— *	*0.3293 *	*M400003416 *
*— *	*0.329 *	*M400005662 *
*Slc25a30 *	*0.3283 *	*M200013505 *
*Akr1c6 *	*0.3158 *	*M400000447 *
*9930111J21Rik *	*0.3155 *	*M400009499 *
*Zfp277 *	*0.3129 *	*M400008100 *
*— *	*0.3083 *	*M400008356 *
*— *	*0.3009 *	*M400005940 *
*Cyp4f18 *	*0.2936 *	*M400011465 *
*— *	*0.2926 *	*M400008231 *
*— *	*0.2772 *	*M400005702 *
*Cyp4f18 *	*0.2688 *	*M400000072 *
*Stard5 *	*0.249 *	*M200005178 *
*— *	*0.2357 *	*M400012678 *
*Tap2 *	*0.2245 *	*M200003350 *
*— *	*0.2197 *	*M400008304 *
*— *	*0.1969 *	*M400005144 *
*— *	*0.1619 *	*M400012679 *

The different expression genes between the POF group and the WT group. There are 90 genes, 33 genes upregulated and 57 genes downregulated. Bold: upregulated genes. Italic: downregulated genes.
